# Pool vs single sample determination of serum prolactin to explore venipuncture associated stress induced variation

**DOI:** 10.1038/s41598-022-27051-8

**Published:** 2023-01-03

**Authors:** Madhumita Das, Chitralekha Gogoi

**Affiliations:** 1 Guwahati Neurological Research Centre Medical Lab, North Guwahati, 781031 India; 2 Guwahati Neurological Research Centre Lab Services, Sixmile, Guwahati, 781022 India

**Keywords:** Hormones, Peptide hormones, Biochemistry

## Abstract

Stress is identified as a cause of transient hyperprolactinemia, whereas venipuncture is considered a source of stress for patient. The aim of this study was to investigate the association of venipuncture-induced stress with elevation of serum prolactin. This was a cross-sectional observational study conducted on a group of 150 outdoor patients visiting a tertiary care hospital. Serial sampling was performed by drawing venous blood at different time intervals (0, 30 and 60 min) by single venipuncture to measure serum prolactin to diagnose stress-induced hyperprolactinemia. The study was conducted in two phases, namely, Phase 1 and Phase 2, at different times. The Phase 1 results were divided into two groups: Group 1 (0 min) and Group 5 (pool prepared from samples collected at 0 + 30 + 60 min). Likewise, the results of Phase 2 were segregated into five groups; Group 1 (0 min), Group 2 (30 min), Group 3 (60 min), Group 4 (average of three groups), and Group 5 (pool from samples collected at 0 + 30 + 60 min). In both Phase 1 and Phase 2 of the study, there was a statistically significant (*p* = 0.0003 in Phase 1 and *p* = 0.02 in Phase 2) decrease in the mean prolactin (17.99 ± 24.76 ng/mL in Phase 1 and 19.61 ± 23.42 ng/mL in Phase 2) in the pooled samples (Group 5) in comparison to the mean prolactin (19.67 ± 27.69 ng/mL in Phase 1 and 21.06 ± 25.06 ng/mL in Phase 2) of the serum collected at 0 h (Group 1). There was no significant difference in the mean prolactin measured from the pooled samples and average prolactin calculated after individual testing from each sample collected at 0 h, 30 min and 60 min. Venipuncture-triggered fear and apprehension may result in transient hyperprolactinemia. In comparison to performing multiple testing on the samples collected at different time intervals and determining the mean, measurement of the analyte from the pooled serum is the better alternative as it can conserve both time and resources.

## Introduction

Prolactin (PRL) is a peptide hormone secreted from acidophilic lactotroph cells of the anterior pituitary gland^1,2^. PRL secretion is intermittent and under neuro-endocrinal control mainly through the prolactin releasing factor (PRF) and prolactin inhibiting factor (PIF)^3,4^. Secretion of prolactin does not adhere to the typical circadian rhythm but follows a characteristic short-term ‘pulsatile’ pattern. It has been determined by different studies that a significant number of the patients whose PRL level is found to be elevated in a single test later have normal PRL levels. Although this is usually attributed to stress or medication, it must be noted that PRL secretion displays diurnal variation with a nocturnal peak in the late-night/early morning hours. Circadian rhythm could be changed with puberty or adult physiology^2,5–12^. Thus, to minimize the effect of pulsatility, the measurement of the serum PRL level may be performed using 2–3 samples collected at 15–20 min intervals^6,13^. However, according to the Endocrine Society Clinical Practice Guidelines, only one elevated serum PRL level (i.e. above the upper limit of the normal range) is sufficient to confirm the diagnosis of hyperprolactinemia, provided the sample is collected without significant venipuncture stress^6^. Usually the optimal time for the collection of blood sample is 2–3 h after waking. Although a blood PRL level greater than 25 ng/mL is considered hyperprolactinemia, a mildly elevated PRL level (20–40 ng/mL) must be confirmed twice to avoid over diagnosis caused by transient elevation of the serum PRL level triggered by certain physiological and psychological factors^2,14,15^.

Numerous factors regulate the PRL secretion. It is primarily under inhibitory control of the hypothalamus, mediated through dopamine (catecholamine), which is the key PIF^11–13,16,17^. PRL secretion is also inhibited by acetylcholine, oxytocin, vasopressin, vasoactive intestinal peptide, pituitary adenylate cyclase-activating peptide, angiotensin II, neurotensin, neuropeptide Y, calcitonin, bombesin like peptides, atrial natriuretic peptide, and prolactin itself through an excitatory mechanism of the dopamine neurons^13,18^. Furthermore, PRL secretion is stimulated by thyrotrophin releasing hormone (TRH), vasoactive intestinal polypeptide, serotonin, noradrenaline, histamine, galanin, somatostatin, cholecystokinin, ɣ amino butyric acid (GABA), nitric oxide, oestrogen, oestradiol, endogenous opioids etc^11–13,19,20^. Reports related to the effect of medication and physiological factors in PRL levels are available. Drugs, such as phenothiazine, also increase PRL secretion^1,21^. By 1970s, stress was identified as a cause of transient hyperprolactinemia, and venipuncture was considered to be a source of stress in the patient^22–25^. Previous studies have reported that the stress-induced variation of neuroendocrine, i.e. dopamine and serotonin, is the basis of prolactin release, which causes functional hyperprolactinemia^2,26^. Stress, whether psychological or induced by illness, surgery, anesthesia, exercise, etc. lead to physiological elevation of the serum PRL levels several-fold^1,7,12,27^. Serial blood sampling by drawing blood samples at 0, 30 and 60 min was considered to be effective in diagnosing stress-induced hyperprolactinemia^2,28^. However, serial blood sampling after 15 min of rest period was also attempted as a measure to correct stress-induced hyperprolactinemia^2,22^.

The primary objective of the current study is to explore plausible stress-induced variation in serum prolactin concentrations.

## Materials and methods

The present study was conducted on a total 150 participants after obtaining written informed consent from them at Guwahati Neurological Research Centre (GNRC) from 1st July 2019 to 31st June 2021, in accordance with the relevant guidelines and regulations, and approved by the Institutional Ethical Committee. All the participants enrolled in the study were selected from the outpatient department, provided they fulfilled the criteria for inclusion. Although relevant information about the relationship between the menstrual cycles and sampling day of the women, (who constituted almost the entirety of the study) could not be ascertained, majority were in their reproductive age group. However, patients using antidepressants, oral contraceptive pills, and other medications and pregnant and lactating women were excluded from the study. As mildly obese females have an enhanced PRL secretion across the 24-h cycle in comparison with a normal female, only females with a body mass index (BMI) between 18.5 and 24.9 (which is considered the healthy range) were included in the study. The BMI was calculated using a standard formula (BMI = kg/m^2^, where kg is the weight of the subject in kilograms and m^2^ is their height in meters squared)^29^. General emotional stress and anxiety associated with blood collection were eliminated by performing the sampling in a silent comfortable room. As hypoglycemia has been reported to acutely stimulate PRL secretion, fasting samples have not been preferred for this study^30^. Perceived stress scale (PSS), the most widely used psychological instrument for measuring the perception of stress, was used to measure the stress level of the patients^31^. The scale of perceived stress was applied before the blood sampling. On arrival, the participants were allowed to sit for 15–20 min to rest and meanwhile asked to fill-up the questionnaires for the PSS to assess the stress level. The scale includes ten simple direct questions about feelings and thoughts in the past month. For better understanding, the results of the PSS scoring were divided into 3 subgroups: Group A, Group B, and Group C, where

- Group A represents the PSS score in the group of patients with elevated serum PRL levels in the subsequent samples,
- Group B represents the PSS score in the group of patients with decreased serum PRL levels in the subsequent samples, and
- Group C represents the PSS score in group of patients in whom the serum PRL levels remain unchanged in the subsequent samples.

The recommended sample type for PRL estimation is serum. As per the Clinical and Laboratory Standards Institute (CLSI) guidelines, samples were collected in a gel vacutainer observing universal precautions for venipuncture. Two mL of blood were collected in the gel vacutainer according to the aseptic venipuncture procedure followed by two more samplings (2 mL each time) at half-hour intervals. A peripheral intravenous cannula was used to draw the sample such that with a single venipuncture, three samples could be collected.

Three samples were collected in total, at 0, 30 and 60 min. The blood samples were centrifuged 30 min after collection (time for clot retraction) at 3000 × *g* for 5 min followed by analysis. Subsequently, the serum was used to measure the PRL level. If the assay was not performed within 8 h of the collection of the samples, the specimens were stored at 2–8 °C till the next day. The serum PRL was measured by a two-site sandwich direct chemiluminometric technique in the ADVIA Centaur CP platform (Siemens Healthcare Diagnostics, Germany).

To monitor the system performance, intra and inter assay reproducibility were checked using the three levels (Level I, Level II, and Level III) of the commercially-available quality control (QC) materials from Bio-Rad. Arithmetic mean (X), standard deviation (SD), and coefficient of variation (CV) were calculated from the individual results of the control series and used to verify the system performance. The CVs of the intra and inter assay reproducibility were < 6.0% all throughout the study period. The samples were analyzed only after obtaining a satisfactory level of performance that is the QC values were within the laboratory calculated range as determined by an internal QC program.

The reagents used for the determination of the serum PRL level were procured from Siemens Healthcare Diagnostics, Germany. The reagents were calibrated at a frequency of 28 days with a two-point calibrator using a master curve calibration. Additionally, recalibration was performed with every new batch of reagents and when there was QC failure.

For determination of the serum PRL, two separate antibodies were used. One was a polyclonal goat anti-prolactin antibody labeled with acridinium ester present in the Lite reagent and the other was a solid phase monoclonal mouse anti-prolactin antibody covalently bound to paramagnetic particles. The PRL present in the serum first combined with the polyclonal goat anti-prolactin antibody to form a prolactin-anti-prolactin antibody complex, which then combined with the solid phase monoclonal mouse anti-prolactin antibody; excess antibody was removed from the reaction mixture by thorough washing. Subsequently, acid and base reagents were used to initiate the chemiluminescent reaction. The amount of relative light units (RLU) generated by the system is directly proportional to the amount of PRL present in the patient sample. If a result exceeded the detection limit (200 ng/mL), automatic dilution (up to 2 and 5 dilution factors) of the sample was performed using ADVIA Centaur Multi-Diluent 1, followed by retesting and calculating the value after adjusting the dilution factor.

### Statistical analysis

For standard statistical analysis, Microsoft Office Excel Worksheet and Graph Pad Prism 5 software were used. Paired *t*-test was used for the statistical analysis where, statistical significance was denoted by ‘*’ (*p* < 0.05), ‘**’ (*p* < 0.01), ‘***’ (*p* < 0.001), and ‘****’ (*p* < 0.0001). The results were expressed as mean ± SD.

### Ethical approval

Study was conducted in accordance with relevant guidelines and regulations and approved by Institutional Ethical Committee i.e. INSTITUTE OF NEUROLOGICAL SCIENCES ETHICS COMMITEE (ECR/778/Inst/AS/2015/RR-18) approved the study, Vide Ref No: Inst/AS/2015/RR-2018/EC-157.

## Results

The objective of the current study was to measure the serum PRL collected at three different time intervals, that is, at 0 h, after 30 min and after 60 min, to evaluate the venipuncture stress-induced variation of the serum PRL level. However, owing to some technical difficulties involved, the study criteria could only be applied to 60 participants. Therefore, determination of the serum PRL was achieved in two phases. In Phase 1, upon determining the serum PRL level for the samples collected at 0 h, all the samples (i.e. serum collected at 0 h, after 30 min, and after 60 min) were mixed to prepare a pool, and PRL was determined from the pooled serum. In Phase 2, in addition to measuring the PRL from the pooled samples, individual analysis was also conducted for each sample collected at 0 h, after 30 min, and after 60 min. A total of 150 participants were incorporated in Phase 1 and 60 in Phase 2 study. For the convenience of data analysis, the results of serum PRL levels obtained from the patients were categorized into five Groups.

- Group 1: Serum PRL levels for the samples collected at 0 h.
- Group 2: Serum PRL levels for the samples collected after 30 min.
- Group 3: Serum PRL levels for the samples collected after 60 min.
- Group 4: Average serum PRL levels of the above three groups.
- Group 5: Serum PRL levels obtained from the pooled samples.

The ages of the selected participants of the current study were dispersed, ranging from 11 to 71 years. However, the predominant age group was 16 to 45 years (Fig. S1a,b, refer to Supplementary File), comprising mostly females with a male to female distribution ratio of 3:97 (Fig. S1c,e, refer to Supplementary File) in both the study groups. The calculated BMI of the study group was approximately 21 (Phase 1 = 20.83 ± 1.68 and Phase 2 = 21.07 ± 1.93) (Fig. S1d, refer to Supplementary File). As the results of both the study groups did not follow a normal distribution (Fig. S1f,g, refer to Supplementary File), we applied log transformation of the data for statistical comparison, following which a normal distribution of the results was observed (superimposed red bell-shaped curve over the histogram in Fig. S1f,g, refer to Supplementary File).

**Figure 1:**
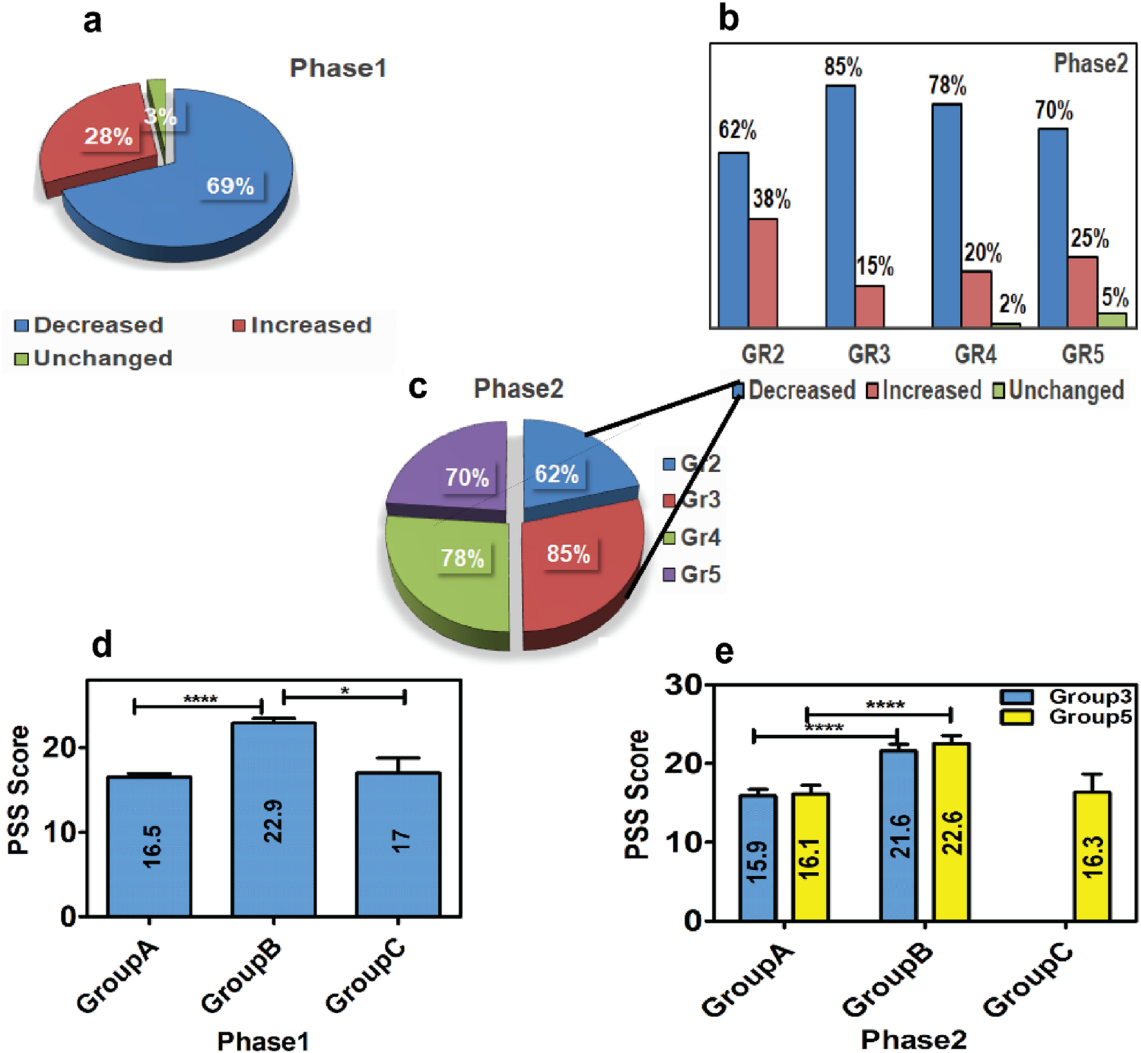
Graphical Illustration of the (**a**) number of participants (in percentage) showing variation in serum prolactin (PRL) level in the pooled samples of Phase 1 study, (**b**) number of participants (in percentage) showing variation in PRL level in different groups of Phase 2 study, and (**c**) number of participants showing drop in serum PRL level in different study groups of Phase 2 study. (**d**) Calculated PSS score of the patients of Phase 1 and (**e**) Phase 2 study, where Group A represents the PSS Score in group of patients with elevated serum PRL level in the subsequent samples; Group B represents the PSS score in group of patients with decreased serum PRL level in the subsequent samples, and Group C represents the PSS score in the group of patients in whom serum PRL level remains unchanged in the subsequent samples. [Results are expressed in mean ± SD; statistical significance denoted by ‘*’ corresponds to *p* value of < 0.05 and ‘****’ corresponds to *p* < 0.0001].

In the present study, 69% (Fig. 1a) of the participants in Phase 1 study and 62–85% (Fig. 1b) of the participants in Phase 2 study exhibited a drop in the serum PRL level in the subsequent samples compared to the first sample. In Phase 2 study, a decrease in the PRL level was observed in 85% of the participants in Group 3 (Fig. 1c), which was higher than that observed in other groups. In Phase 1 study, the mean PSS scores of Group A, Group B and Group C were 16.5 ± 2.9, 22.9 ± 5.8, and 17 ± 3.6, respectively. It was significantly high in Group B (*p* ˂ 0.0001) compared to Group A. The difference was also significant between Group B and Group C (*p* = 0.03) (Fig. 1d, Table S1, refer to Supplementary File). Similar findings were also observed in Phase 2 study, where in Group 3, the mean PSS score was 15.9 ± 2.5 for Group A and 21.6 ± 6.2 for Group B, and the difference was highly significant with a *p* value of ˂ 0.0001. No data was available for Group C. In Group 5, the mean PSS score was 16.1 ± 4.1 for Group A, 22.6 ± 6.8 for Group B, and 16.3 ± 4.0 for Group C. The PSS score calculated was significantly higher (*p* ˂ 0.0001) in Group B compared to that in Group A (Fig. 1e, Table S1, refer to Supplementary File).

**Figure 2:**
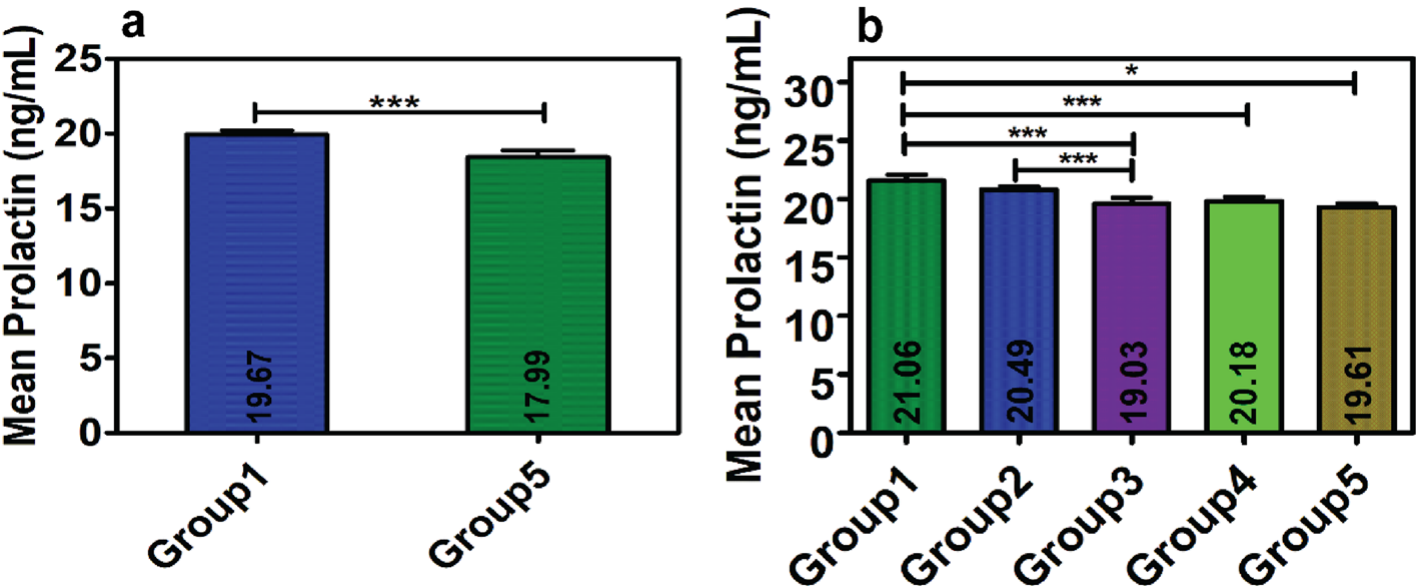
Distribution of mean serum prolactin levels and statistical comparison for different groups in (**a**) Phase 1 and (**b**) Phase 2 study. [Results are expressed as mean ± SD; statistical significance denoted by ‘*’ corresponds to *p* value of < 0.05 and ‘***’ corresponds to *p* < 0.001].

The results of Phase 1 study (Table 1 and Fig. 2a) showed a wide range of distribution for serum PRL from 1.09 to 200 ng/mL in both the studied groups. The observed mean serum PRL level (17.99 ± 24.76 ng/mL) from the pooled samples (Group 5) decreased by 1.68 ng/mL when compared to the mean PRL level (19.67 ± 27.69 ng/mL) of the serum collected at 0 h (Group 1). It was observed that the mean difference of the compared groups was statistically significant for the paired *t* test with a “*p* value of 0.0003” at the *t* value of 3.68.

**Table 1:** Results along with statistical analysis (paired *t*-test) of serum prolactin level in different groups in Phase 1 study.

Prolactin level (ng/mL)	At 0 h (Group 1)	Pooled serum (Group 5)	Statistical parameters
Mean ± SD	19.67 ± 27.69	17.99 ± 24.76	Difference = 1.68
Minimum	1.09	1.09	*t* value = 3.68
Maximum	200	200	*p* value = 0.0003 (***)

The serum PRL levels of the different groups in Phase 2 were found to be distributed in a narrow range, varying from 1.0 to 95.4 ng/mL, compared to that in Phase 1 (Group 1 = 1.09–95.4 ng/mL, Group 2 = 1.0–93.66 ng/mL, Group 3 = 1.2–94.1 ng/mL, Group 4 = 1.1–94.23 ng/mL, and Group 5 = 1.09–95.4 ng/mL) (Table 2). The results showed a decreasing trend in the serum PRL level in the samples collected after 30 min (Group 2) and 60 min (Group 3), in contrast to the samples collected at 0 h (Group 1). The calculated arithmetic mean for Group 1, Group 2, and Group 3 was 21.06 ± 25.06, 20.49 ± 24.05, 19.03 ± 23.30 ng/mL, respectively (Table 2).

**Table 2:** Results of serum prolactin level for different groups in Phase 2 study.

Prolactin (ng/mL)	Group 1 (n = 60)	Group 2 (n = 60)	Group 3 (n = 60)	Group 4 (n = 60)	Group 5 (n = 60)
Mean ± SD	21.06 ± 25.06	20.49 ± 24.05	19.03 ± 23.30	20.18 ± 24.04	19.61 ± 23.42
Minimum	1.09	1.0	1.2	1.1	1.09
Maximum	95.4	93.66	94.1	94.23	95.4

The statistical analysis of the data revealed significant deviation in the mean value between the different groups (Table 3 and Fig. 2b).

**Table 3:** Results of statistical analysis (paired *t* test) between different groups of Phase 2 study.

Prolactin (ng/mL)	Gr1-Gr2	Gr1-Gr3	Gr2-Gr3	Gr1-Gr4	Gr1-Gr5	Gr4-Gr5	Gr3-Gr4	Gr3-Gr5
Difference	0.57	2.03	1.46	0.88	1.45	0.57	− 1.15	− 0.58
*t* Stat	1.62	4.13	3.64	3.5	2.34	1.37	− 0.26	− 0.13
*p* value	0.68 (ns)	0.0001 (***)	0.0005 (***)	0.0008 (***)	0.02 (*)	0.17 (ns)	0.79 (ns)	0.89 (ns)

*Gr* group.

A drop in the mean PRL by 2.03 ng/mL was observed in Group 3 in comparison to that in Group 1 (Table 3), which was highly significant with a *p* value of 0.0001 and *t* = 4.13. However, the decrease (0.57 ng/mL) in Group 2 was not statistically significant (*p* = 0.68 with *t* = 1.62). Upon comparing the mean, a highly significant decrease was observed in Group 3 compared to that in Group 2, with a difference of 1.46 ng/mL (*p* = 0.0005 and *t* = 3.64). The results for Group 4 and Group 5 did not exhibit much variations, presenting arithmetic means of 20.18 ± 24.04 ng/mL (Group 4) and 19.61 ± 23.42 ng/mL (Group 5), respectively, with a difference of 0.57 (*t* = 1.37 and *p* = 0.17). However, a significant decrease in the mean values of Group 4 and Group 5 was observed compared to that of Group 1 (*p* = 0.0008 with *t* = 3.5 between Group 1 and Group 4; *p* = 0.02 with *t* = 2.34 between Group 1 and Group 5). The mean PRL of Group 3 did not show a significant variation from that of Group 4 and Group 5.

 A total of 119 participants in Phase 1 study presented with normal PRL levels (≤ 25 mg/ml) with a mean of 9.72 ± 4.41, which subsequently decreased in the pooled serum samples to 9.2 ± 4.32, albeit statistically insignificant (*p* = 0.36). However, the remaining 31 patients presented with hyperprolactinemia with a mean PRL of 57.85 ± 42.8, which then significantly reduced to 51.75 ± 38.56 (*p* = 0.005). Of these 31 hyperprolactinemia patients, two (6.5%) had presented with normalized PRL during testing. As a total of only seven patients revealed moderately high PRL, (i.e. results between 25 and 35 ng/mL) with a mean of 31.45 ± 3.9, which significantly reduced to 28.84 ± 3.08 (*p* = 0.05) during testing, the possibility of obtaining more normalized results existed in case of increased data in the said group. Likewise, in Phase 2 study, 48 participants presented normal PRL levels (mean = 9.81 ± 4.11), and the remaining 12 displayed hyperprolactinemia (mean = 66.04 ± 23.17), of which one patient (8.3%) presented normalized PRL during testing (Table 4). However, the occurrence of substantially few observations in Phase 2 study might present difficulties in obtaining a proper correlation and statistically significant result.

**Table 4:** Results and statistical comparison of the normal and high results of both Phase 1 and Phase 2 study.

Study	Prolactin level	n	Mean ± SD		Difference	*t* value	*p* value
			Group 1	Group 5			
Phase 1 (n = 150)	Normal	119	9.72 ± 4.41	9.2 ± 4.32	0.52	0.92	0.36
	High	31	57.85 ± 42.8	51.75 ± 38.56	6.1	3.04	0.005
	Moderately high (25–35)	07	31.45 ± 3.9	28.84 ± 3.08	2.61	2.36	0.05
Phase 2 (n = 60)	Normal	48	9.81 ± 4.11	9.36 ± 3.95	0.45	0.55	0.59
	High	12	66.04 ± 23.17	60.61 ± 24.14	5.43	1.89	0.08
	Moderately high (25–35)	02	30.76 ± 3.27	31.25 ± 2.59	–	–	–

As a testing pool was prepared for analysis in Phase 1 and no statistical difference was observed among the results of Group 3 (60 min collection), Group 4 (mean of 0 + 30 + 60 min collections), and Group 5 (pooled serum) in Phase 2, the results of Group 1 were statistically compared with that of Group 5 only.

The differences in the mean serum PRL level between different study groups (Phase 2 study) were represented in a pie diagram (Fig. S2, refer to Supplementary File). The maximum difference was observed between Group 1 and Group 3 (29%), and the difference between Group 1 and Group 2 and that between Group 4 and Group 5 were found to be minimal (8%).

**Figure 3:**
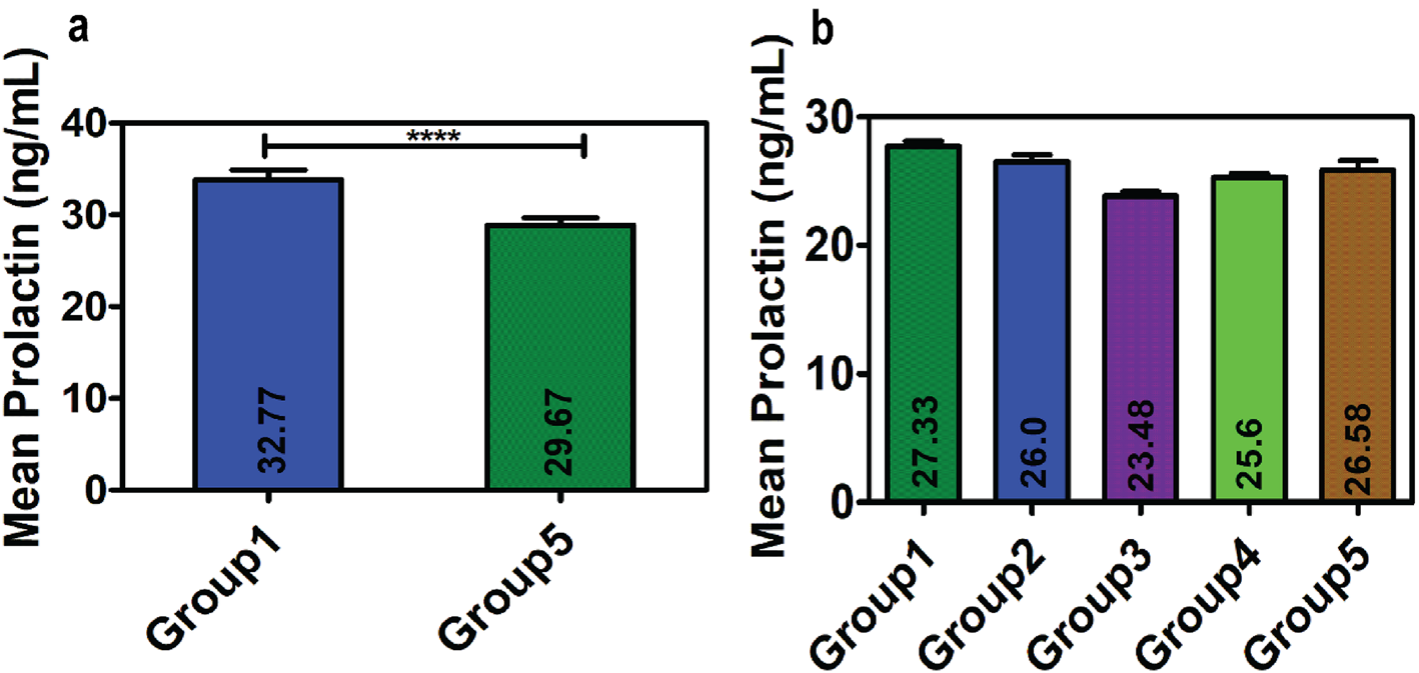
Graphical representation of the results between 20 and 40 ng/mL for the different groups in (**a**) Phase 1 and (**b**) Phase 2 study. [Results are expressed as mean ± SD; statistical significance denoted by ‘****’ corresponds to *p* < 0.0001].

Nevertheless, a serum PRL value higher than 25 ng/mL is considered as hyperprolactinemia; it is recommended that borderline cases (20–40 ng/mL) are verified twice before reporting to avoid over diagnosis. Therefore, borderline cases were analyzed separately to determine if transient hyperprolactinemia resulted from the venipuncture-induced stress. In Phase 1 study, the mean serum PRL of the borderline cases (mean of serum PRL values between 20 and 40 ng/mL) of Group 1 (32.77 ± 5.49) significantly dropped to 29.67 ± 4.81 ng/mL, which was the mean serum PRL obtained for Group 5, with a difference of 3.1 ng/mL and *p* value of < 0.0001 (Table 5 and Fig. 3a).

**Table 5:** Statistical analysis (paired *t* test) of the results between 20 and 40 ng/mL for the different groups in Phase 1 and Phase 2 study.

Phase 1 (n = 21)			Phase 2 (n = 03)				
Parameters	Group 1	Group 5	Group 1	Group 2	Group 3	Group 4	Group 5
Mean ± SD	32.77 ± 5.49	29.67 ± 4.81	27.33 ± 6.38	26 ± 7.87	23.48 ± 9.02	25.6 ± 7.74	26.58 ± 8.29
Difference with Group 1		3.1		1.33	3.85	1.73	0.75
*t* value		5.32		1.38	2.29	1.96	0.59
*p* value		< 0.0001		0.3	0.1	0.1	0.6

In Phase 2 study, the marginally high mean value of Group 1 (27.33 ± 6.38) dropped to a normal level (23.48 ± 9.02) in Group 3 (collected after 60 min rest) (Table 5 and Fig. 3b). Although statistically insignificant, there was a considerable decrease in the mean PRL in all the subsequent groups compared to that in Group 1, indicating the possibility of over diagnosis owing to transient elevation of the serum PRL level because of venipuncture-induced stress. However, the results might have been compromised as the number of observations was very less (3) in Phase 2 study, preventing proper interpretation.

## Discussion

The present study was designed to explore the variation in the serum PRL level in response to venipuncture-induced stress. PRL estimation is mired in many controversies in relation to the ideal practice of sample collection^32^. The fear and apprehension triggered by venipuncture have been reported to induce transient hyperprolactinemia. As a result of the multifaceted physiological response to stress, there is release of epinephrine and norepinephrine accompanied by the activation of hypothalamic–pituitary–adrenal axis, thus leading to PRL secretion^2,33^. The prevalence of functional hyperprolactinaemia in response to clairvoyant stress, which may be caused by hospital attendance or venipuncture, is not adequately explored. In the present study, we collected three blood samples at intervals of 30 min each to minimize the fear and anxiety factor by allowing a break for rest and relaxation along with counselling.

In both the study groups (Phase 1 and Phase 2), the group of patients showing reduced PRL level in the subsequent samples exhibited significantly high PSS score compared to the group of patients who did not exhibit any reduction in the serum PRL level (either increased or remained same) in the subsequent samples, indicating the possibility of stress-induced hyperprolactinemia.

In the current study, a drop in the serum PRL levels was observed in the subsequent samples of more than 60% of the participants in both Phase 1 and Phase 2 compared to that in participants of Group 1 (collected at 0 h), substantiating the association of venipuncture-induced stress with increased PRL secretion. As reported in several previous studies^22,24,28^ where the initial raised serum PRL level decreased in subsequent samplings, we also observed that reduction of stress and anxiety with time and rest resulted in decreased PRL secretion. The evidence of the statement was the results of Group 3 (collected after 60 min rest) in Phase 2 study, where the maximum number of participants (85%) exhibited a significant drop in the serum PRL levels. Moreover, in both Phase 1 and Phase 2 study, a statistically significant decrease in the mean PRL level was observed in the subsequently collected samples. In Phase 1 study, the mean serum PRL in the pooled sample decreased by 1.68 ng/mL (*p* = 0.0003) from the mean PRL level of 19.67 ± 27.69 ng/mL of the first samples (0 h) to 17.99 ± 24.76 ng/mL of the pooled samples. Similarly, in Phase 2 study, a statistically significant drop in the mean serum PRL level was observed in all the study groups compared to that in Group 1, excluding Group 2 where the drop in the mean value was insignificant. Maximum reduction in the mean serum PRL level was observed in Group 3, wherein it decreased by approximately 2.03 ng/mL compared to that in Group 1. It was also observed that during testing, the mean of both the moderately high and high results were found to be significantly decreased in Phase 1 study. This decrease was not significant in Phase 2 study as proper statistical comparison was not possible because of the limited quantity of data available in Phase 2 study.

This confirmed that there was adequate relaxation to cause release of stress, which was sufficient to result in a decrease in the PRL secretion. In some previous studies, in order to minimize the venipuncture-induced stress, attempt was made to introduce an intravenous catheter for serial sampling^22^. However, introducing a catheter in more stressful than venipuncture, which may be a factor behind the insignificant drop in the PRL level even after rest. Few experiments were conducted where serial sampling was practiced at 15 min intervals^22,32^. The observed insignificant drop in the mean serum PRL level after 15 min rest^32^ demonstrates that ample time is required to reduce the fear, apprehension, and stress and to relax the patient. In the present study, the insignificant drop (0.57 ng/mL) in the mean serum PRL level in Group 2, (represented by only 8% when compared with the drop in the other groups) adequately explained that 30 min rest and relaxation is not sufficient to reduce the fear and apprehension associated with venipuncture or hospital attendance. Similar findings were also observed in a previous study where there was significant fall in the average PRL of three collections (0 h, 20 min and 60 min) compared to the PRL collected at 0 h. Additionally, significant fall in the PRL was observed after 60 min rest compared to that after 20 min rest^34^. However, in another study where 40 min rest was provided, significant lowering of the PRL was observed^24^. As the results of our study suggested that 30 min rest was not sufficient to lower the venipuncture induced-stress and the mean PRL of Group 3 did not show any statistically significant difference with those of Group 4 and Group 5, two collections (one at 0 h and second after 60 min rest) and the measurement of serum PRL from the pooled serum were considered as preferred alternatives to negate the possibility of false elevation from venipuncture-induced stress.

Few researchers have recommended reporting the average of the three results obtained from the three samples collected at 15 min intervals as a measure to minimize false reporting of hyperprolactinemia resulting from venipuncture-induced stress^22,32^. However, our study revealed that there was no significant difference between the average of the three individual results obtained from the three samples collected at 30 min intervals (Group 4) and Group 5 results (pooled serum). Thus, it can be concluded that a single measurement from the pooled serum is sufficient for interpretation, which is preferred over wasting resources by three separate measurements. In accordance with our findings, some previous studies also recommended multiple sampling and estimation of serum PRL from a pooled sample to minimise over diagnosis and wastage of laboratory resources^11,34,35^.

Since there was a noticeable drop in the serum PRL after rest, the chances of over diagnosis, particularly, in case of patients with mildly elevated serum PRL cannot be ruled out. Moreover, in our study, it was observed that borderline cases exhibited a significant drop in the PRL level (by 3.1 ng/mL with *p* value of < 0.0001 in Phase 1 study) after rest, implying the possibility of over diagnosis. In Phase 2 study, the marginally high mean value of Group 1 (27.33 ± 6.38) dropped to a normal level (23.48 ± 9.02) in Group 3. However, as the number of observations was significantly low in Phase 2, statistical comparison was not able to obtain a statistically significant result.

## Conclusion

An increase in the PRL level was observed in response to venipuncture triggered fear and apprehension, and a period of 60 min rest and relaxation resulted in significant reduction in the serum PRL level. From the results of the present study, it can be concluded that rather than performing multiple tests using samples collected at different time intervals and determining their mean, measurement of the analyte from the pooled serum is the better alternative as it can conserve both time and resources.

## Limitations of the study

Our study has limitations beyond its retrospective view of observation. Transient elevation of the serum PRL level caused by certain physiological and psychological factors do occur; however, collecting multiple samples in clinical practice constitutes the major problem. The decision to submit the patient to a PRL repeated sampling was made by the laboratory. The rationale behind the decision cannot be inferred from clinical records, and therefore, the possibility of a selection bias cannot be ruled out. PRL sampling would more likely be proposed to patients with higher clinical suspicion of stress hyperprolactinemia. In the current study, there was a skewed gender distribution of the study population, the statistical inference for which could not be depicted in regards to gender related stress-induced hyperprolactinemia. The small sample size of the Phase 2 study was sufficient enough to draw statistical conclusions; however, larger sample size with equal gender distribution could have yielded better statistical inferences. Borderline cases could not be identified before analysis, and convincing the patients for repeat testing in a hospital setup was a difficult task. Hence, in such situations, the judicious use of testing modalities, such as MRI, could have been exercised. Technical difficulties directed the study criteria to be applied to 60 participants only. Therefore, the determination of the serum PRL was achieved in two phases. For the convenience of data analysis, the results of the serum PRL levels obtained from the patients were categorized into five Groups. The grouping and regrouping of the participants rendered the study slightly laborious and confusing, which is another limiting factor of the study.

## Supplementary Information

Supplementary Information.

## Data Availability

The authors declare that the data supporting the findings of this study are available within the article [and its supplementary information files].

## Abbreviations

GNRC: Guwahati neurological research center
PRL: Prolactin
PRF: Prolactin releasing factor
PIF: Prolactin inhibiting factor
TRH: Thyrotrophin releasing hormone
GABA: ɣ Amino butyric acid
CLSI: Clinical and laboratory standards institute
QC: Quality control
SD: Standard deviation
CV: Coefficient of variation
RLU: Relative light units
BMI: Body mass index
PSS: Perceived stress scale
