# Supplementary material for: Pool vs single sample determination of serum prolactin to explore venipuncture associated stress induced variation
Source: Sci Rep. 2023 Jan 3;13:113. doi: 10.1038/s41598-022-27051-8 (PMC9810589; doi:10.1038/s41598-022-27051-8)
Supplement: Supplementary file 1 — Supplementary Information. [file 41598_2022_27051_MOESM1_ESM.pdf]

SUPPLEMENTARY FILE

Pool vs Single Sample Determination of Serum Prolactin to Explore Venipuncture Associated Stress Induced Variation

Authors: Dr. Madhumita Das[1] MD, PhD; Dr. Chitralekha Gogoi[2] MD

Affiliation: [1]Guwahati Neurological Research Centre Medical Lab, North Guwahati, 781031

[2]Guwahati Neurological Research Centre Lab Services, Sixmile, Guwahati, 781022

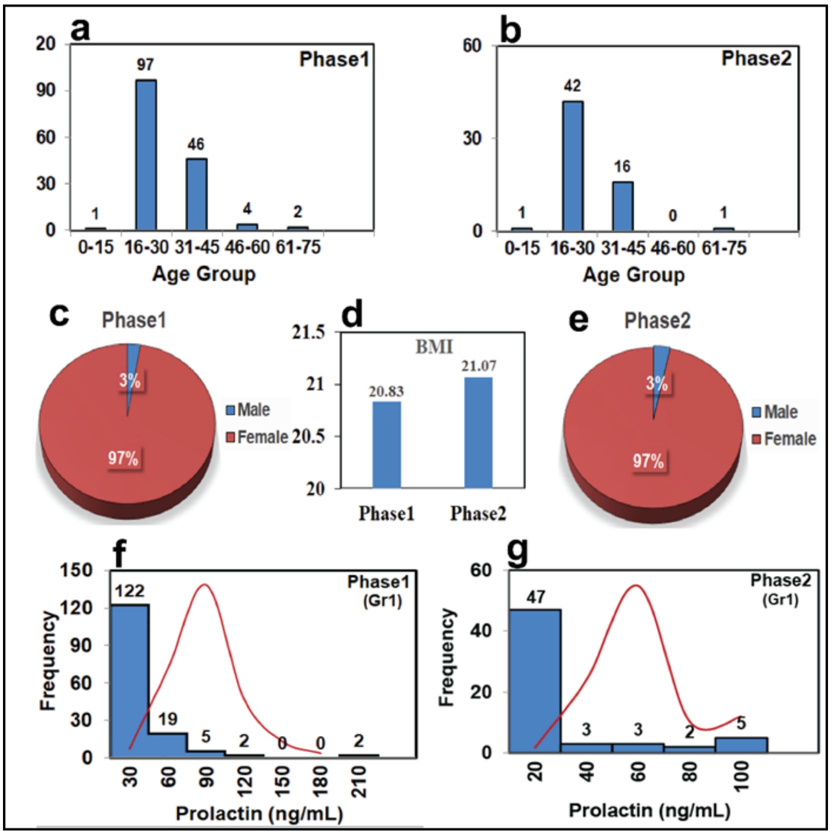

FigureS1. Illustrative demonstration of age (a,b); gender distribution (c,e); and BMI (d) of both Phase1 and Phase2 study along with distribution of serum prolactin level of Phase1(f) and Phase2 (g) study (Superimposed red lines represent the distribution of serum prolactin after log transformation of the data).

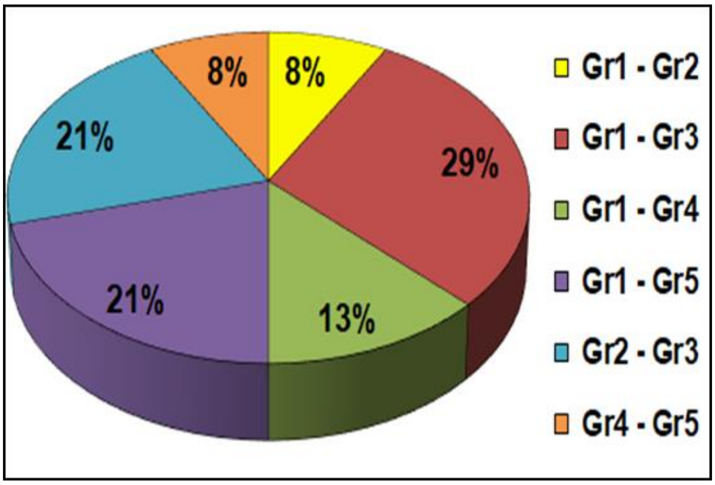

FigureS2. Graphical representation of mean differences of serum prolactin between different groups of Phase2 study.

TableS1. Statistical analysis (Paired *t*-Test) of PSS score of different groups of both Phase1 and Phase2 study

**Study Subjects**			**Mean & SD**	***t* value**	***p* value**	**Number**
**Phase1 Study**	**Group5**	**GroupA**	16.5 ± 2.9			42
		**GroupB**	22.9 ± 5.8	-8.9	< 0.0001	104
		**GroupC**	17 ± 3.6	3.16	0.03	4
**Phase2 Study**	**Group3**	**GroupA**	15.9 ± 2.5			9
		**GroupB**	21.6 ± 6.2	-4.75	< 0.0001	51
	**Group5**	**GroupA**	16.1 ± 4.1			14
		**GroupB**	22.6 ± 6.8	-4.55	< 0.0001	43
		**GroupC**	16.3 ± 4.0	2.49	0.09	3
